# Regional planning for meaningful person-centred care in mental health: context is the signal not the noise

**DOI:** 10.1017/S2045796020000153

**Published:** 2020-02-24

**Authors:** D. Rock, S. P. Cross

**Affiliations:** 1 WA Primary Health Alliance, Perth, Western Australia; 2 Discipline of Psychiatry, University of Western Australia; 3 Brain and Mind Centre, University of Sydney

**Keywords:** Health service research, mental health, performance, quality of care, social factors

## Abstract

Person-centred care is at the core of a value-based health system. Its transformative potential is to enable and support key policy, planning and service developments across the system even when these go against the self-interest of individual major players. It offers a potent test for decision makers at all levels. It demands responses that are multi-level, empirically grounded, expert-informed and data-driven that must converge on the singularity of individuals in the places that they live. This requires different approaches that recognise, respect and reconcile two necessary but constitutionally disparate perspectives: the bureaucratic, overtly decontextualised, top-down, policy and planning objectives of central governments and the formally complex, dynamic and contextualised experience of individuals in the system. Conflating the latter with the former can lead unwittingly to a pervasive and reductive form of quasi-Taylorism that nearly always creates waste at the expense of value. This has parallel application in the treatment domain where outcomes are non-randomly clustered and partitioned by socioeconomic status, amplifying unwarranted variation by place that is striking in its magnitude and heterogeneity. In this paper, we propose that a combination of (1) relevant, local and sophisticated data planning, collection and analysis systems, (2) more detailed person-centred service planning and delivery and (3) system accountability through co-design and transparent public reporting of health system performance in a manner that is understandable, relevant, and locally applicable are all essential in ensuring planned and provided care is most appropriate to more than merely the ‘average’ person for whom the current system is built. We argue that only through a greater appreciation of healthcare as a complex adaptive (eco)system, where context is everything, and then utilising planning, analysis and management methodologies that reflect this reality is the way to achieve genuine person-centred care.

A consistent recommendation arising from countless reviews into the mental health system both within Australia (National Mental Health Commission (Australia), 2014; Department of Health (Australia), 2015) and internationally (Institute of Medicine, 2001; World Health Organization, 2001; Institute of Medicine, 2006; World Health Organisation, 2016) is adherence to the central concept of ‘person-centred care’. The quality of the system as a whole is only meaningful by, and through, the effects on individuals using the system (Berwick, 2002) and the value determined from the users perspective (Porter, 2010). The central challenge for service planners at the local regional level, therefore, is how to unite population-focused policy directives with the context-bound healthcare needs of individuals in local communities.

## Person-centred care is not new age quackery, it is a path to better health for all

While not exactly a new concept (Salvador-Carulla and Mezzich, 2012), person-centred care has been advocated for in a number of subsequent reports providing guidance to mental health system reform (National Mental Health Commission (Australia), 2014; Salvador-Carulla *et al*., 2016). Essentially, person-centred care is an approach that places the person in context, over and above bodily systems or diseases, and places this concept at the centre of healthcare (Mezzich *et al*., 2016). Respecting individual diversity and enabling personal control of healthcare through transparency and system accountability was a foundational recommendation of the Institute of Medicine's (IOM) ground-breaking report ‘Crossing the Quality Chasm’ (Institute of Medicine, 2001), and its mental health companion report ‘Improving the Quality of Health Care for Mental and Substance-Use Conditions’ (Institute of Medicine, 2006). In this regard, there is clear consensus about what needs to change ‘at the coal-face’ to improve healthcare outcomes for individuals and at the broader level, how systems can enable effective pathways, enhance integration, care continuity and collaboration across organisational boundaries and between healthcare professionals (Funk, 2008; Hunt, 2017; Lora *et al*., 2017; Meurk *et al*., 2018). What is not agreed is how this can be achieved at scale across divergent health systems that do not pause operations whilst the required changes are made, nor may not be sufficiently resourced to enact such structural change and operate, in many ways, like pre-industrial organic economies. In the absence of clear, or indeed any, agreement over scalable implementation methodologies, local service planning is undertaken using out-dated models, incentives, data, tools and technologies that reboot the past rather than reframe the future (Duckett, 2008; Rosen *et al*., 2010; Swerissen *et al*., 2016; Looi and Kisely, 2018; Calder *et al*., 2019; Productivity Commission (Australia), 2019). Ultimately, opportunities to improve care for individuals living, as they do, within complex multi-layered social contexts are hidden by what is proposed to work for the average (Fixsen *et al*., 2009).

What is planned for the ‘average’ person with ‘average’ needs does not necessarily translate to what works for unique individuals in the real-world (van Os *et al*., 2019). Achieving true person-centred care requires a change from the usual top-down approaches that struggle against policy dilution and diffusion during implementation where pragmatic decisions progressively decouple what is implemented from what was intended (Sterman, 2001; Carey and McLoughlin, 2016). Thus, it is critical to separate the processes for selecting what is planned to be implemented from the process of implementing what is planned (Greenhalgh *et al*., 2004; Palinkas and Aarons, 2009). Plans that fail to address individuals in their context and the implementation process in an accountable and measurable fashion are fraught from the outset and unlikely to result in effective translation into practice (Aarons *et al*., 2011).

Australia provides an illustrative example. It has a first-world health system where most of the population can access the care they need most of the time (Australian Insitute of Health and Welfare, 2018). Australian governments (federal and state) have multi-level (national, state, region, service, person) mental health policy objectives but lack a corresponding implementation framework to systematically realise these policy ambitions into coordinated and accountable actions that can be undertaken within local geographies to meet the needs of individuals over time in ways that are scalable and comparable (Rosenberg *et al*., 2015). This requires a different planning schema, one that is scalable to context, level and location of the decision-making, incorporates process guidance grounded in implementation science (Nilsen, 2015) that recognises the different information needs of the decision makers across the system (Salvador-Carulla *et al*., 2015; Mezzich *et al*., 2016; Chung *et al*., 2018).

**Fig. 1.**
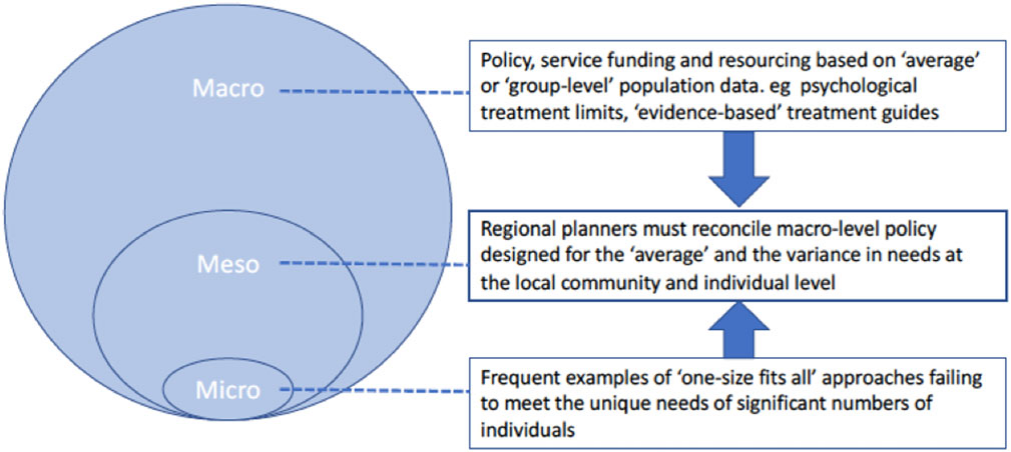
The relationship between ‘meso’ or regional planning level with both the macro (policy) and micro (individual) levels.

New frameworks are required to better connect national policy with individual care experience (Institute of Medicine, 2001; Institute of Medicine, 2006). With this in mind, the IOM proposed four hierarchical levels of all health systems. The first level is the individual or consumer (nano) level; those for whom the health system exists. The second level is the level of small units of care delivery, such as discrete services or individual practitioners. Known as the ‘microsystems of care’ (micro, Fig. 1), it is those that provide the care that consumers directly experience. The third-level (‘meso’, Fig. 1) is that of the larger organisations (such as Primary Health Networks in Australia) that house those smaller units; and the fourth-level (macro, Fig. 1) is that of the broader policy and regulation level. Similar structural hierarchies have also been suggested, such as Tansella and Thornicroft's (1998) which proposes a level (country, local and patient) by phases (inputs, processes and outcomes) matrix approach which guides the functional building blocks for translating policy objectives into nested actions at all levels. Person-centred care ideally plays out at all levels and will look very different at each level (e.g. shared decision-making, service planning and resourcing, policy planning). Thus, it is not the absence of viable frameworks to guide policy to practice translation, but that it requires a normative shift for these to be enabled in practice and at scale in a manner that permits comparison. More tangible connections between (often disjointed) activities at each of the levels must relate in one way or another back to individuals, and not theoretically derived ‘groups’.

Understanding the contextual, structural and unique personal factors that enable individuals to successfully access effective interventions is crucial if we are to realise the benefits of an affordable, accessible, efficient and high-quality health system for all (Salvador-Carulla and Hernández-Peña, 2011). This has practical implications for the planning of healthcare services more broadly, but is critical for the successful operation of local-level care systems where resources are scarce, placing a premium on maximising the technical efficiency of what is available (Duckett, 2008; Fuller *et al*., 2009).

## The current way of planning services is not working locally

Healthcare planning has become increasingly decoupled from reality, replacing this with ‘average man’ models to build wholly personified alt-real worlds that prioritise theoretical frameworks and quantification (of a sort) and downplay or dismiss empiricism and tacit knowledge (clinicians and patients). They are applied top-down in the manner of Soviet-style central planning without consideration of scale or context, with the result they are given lip-service by those responsible for planning, commissioning and managing regional and sub-regional services. The fundamental problem is not ‘should be’ models themselves: there are manifest advantages to be had in the use of such frameworks to estimate and plan resource allocations at the population-level, for example (Department of Health (Australia), 2017; Mental Health Commission (Western Australia), 2019). It is when these models are promoted or applied outside of the spatial, temporal and contextual niches for which they have been constructed that makes them poorly suited to the needs of regional and sub-regional planners, who need to accommodate and incorporate a level of heterogeneity and variation that is necessarily averaged-out in whole-of-population approaches (Aarons *et al*., 2011; Pfadenhauer *et al*., 2017).

Recognising also that a significant proportion of the population do not have access to the personal resources required to enable them to engage with care effectively is essential. Knowing this, it is the responsibility of system managers, not by-default the individual, to build facilitators into the structure of the intervention set itself if we are to develop truly responsive systems of care, not just for some notional average person with average personal resources. The current planning, implementation and service delivery models for mental health treatment in Australia objectively do not adequately meet the breath of needs of individuals or communities.

Access to psychological therapies offers a case in point. Medicare in Australia provides for universal subsidised access to focused psychological therapy via GP referral (Department of Health (Australia), 2019). However, providers can charge any amount above the Medicare schedule fee they wish. The result is significantly greater access and utilisation in locations characterised by higher average socio-economic status, presumably because more individuals in these areas can afford the out-of-pocket expenses (Meadows *et al*., 2015). Not surprisingly then, this results in far higher proportions of available practitioners in these areas. Surely by accident and not design, we have a position where billions of dollars of public funds are spent on primary mental healthcare (Australian Institute of Health and Welfare, 2019) that does not meet either the universality standard (Meadows *et al*., 2015) or minimum adequate levels of provision (Jorm *et al*., 2016), and there is little if any public accountability (Rosenberg *et al*., 2015; Rosenberg and Hickie, 2019).

## Considerations for regional service planners

In addressing (albeit briefly) these issues, we do not suggest that what we offer is a panacea. Instead, we frame a set of priority issues that we face, and in doing so offer tentative recommendations for improvement towards a person-centred mental health service system.

### The missing data thing: operational plans require contextualised data

High-quality operational plans require similarly configured data and analytics to most effectively interpret and apply these to real-world service planning and implementation decisions. Unfortunately, the data required for effective local service planning is in many cases either absent or housed in data silos. The result is a systematically incomplete data landscape and a structural impediment to the delivery of effective and efficient mental healthcare (Whiteford *et al*., 2013). Moreover, there are hidden assumptions within the core conceptual logic of what constitutes a health system that distorts decision-making. We highlight three. First, the systematic excision of informal provision, notwithstanding the fundamental role it has in maintaining overall system viability (Diminic *et al*., 2017). Second, the lack of routine consideration of the dynamic interdependency between the various elements of care, formal and informal that occurs in real-world settings recognising that health systems are complex not simply complicated. This results in category errors and false inferences and the adoption of event-oriented ‘planiverse’ models that inadvertently exclude or ignore contextually important dimensions resulting in poorer outcomes than otherwise might be the case (Sterman, 2001; Atkinson *et al*., 2015; Page *et al*., 2018). Thirdly, the inability to clearly differentiate artificial or failure demand, that is, demand created by the way the system is currently organised and managed, from value demand is a formidable obstacle to effective planning at all levels (Seddon, 2014) as well as confounding attempts at system-level accountability (Fillingham *et al*., 2016). What is required is a departure from simple and linear data analytic methods and an uptake of more sophisticated agent-based data modelling techniques that can deal with complexity and incorporate the full cycle of care in context (Salvador-Carulla *et al*., 2006; Atkinson *et al*., 2015; Salvador-Carulla *et al*., 2016; Page *et al*., 2018). Whilst such approaches are recommended (Cross *et al*., 2019; Hickie, 2019) and the methodologies available (Sterman, 2001; Aarons *et al*., 2011; Atkinson *et al*., 2015), they are not commonly used in routine settings. Clearly, this requires the deployment of updated infrastructure, as well as data collection, analysis and interpretation expertise applied at the local level to ensure contextual factors are incorporated. In this way, a reconciliation of macro-level policy objectives with regional-level (meso-micro) system management can be achieved, which would be a normative shift away from one-size approaches, respecting and leveraging the tacit expertise of residents and practitioners *in situ* (Bate, 2014; Seddon, 2014), recognising it is the people who determine the value of services not the funders (Porter, 2009, 2010).

### Consideration of context in service planning and treatment provision

Treatments conducted in controlled environments and proven efficacious at the group level are the mainstay of ‘evidence-based’ practice; yet these same interventions do not necessarily apply to person-centred care (van Os *et al*., 2019). Indeed, group generalisations always obscure individual difference (Fisher *et al*., 2018), and blindly applying the same interventions to individuals with similar symptom profiles without considering the context is inappropriate.

The disconnect with real-world applicability can be seen with common psychological treatments which are designed, distributed and provided in ways that favour higher socio-economic position (SEP) individuals to a marked greater extent (Meadows *et al*., 2015). Mainstream psychological treatments require active individual engagement, investment in cognitive or behavioural change and enough material resources (formal and informal) if they are to be successful. Further, the psychological sequelae of poverty mean that low SEP individuals are, on average, less able to effectively engage with agentic interventions (Mathers and Schofield, 1998). Indeed, the underpinning agentic assumptions built into most psychological treatment offerings are antithetical to the present-oriented, context-sensitive, functionally adaptive response to deprivation (Henrich *et al*., 2010; Pepper and Nettle, 2017). Thus, we have mainstay treatments that by design work for many but not all, with the result that a significant proportion of the population do not gain access to nominally effective treatments, and overall system demand and costs increase, yet population healthcare outcomes do not improve commensurately (Meadows *et al*., 2018).

This is occurring against a background where current welfare and health policy and practice mean the lower socio-economic strata risk becoming more exclusively composed of individuals and families with consistently fewer personal resources (material and non-material) that increase their risk of poor health outcomes for themselves, and create a toxic legacy for future generations, amplifying rather than attenuating enduring disadvantage (Wilkinson and Pickett, 2009; Calder *et al*., 2019). In Australia at least, these structural inequalities are seemingly hard-wired into the ostensibly universal treatment offerings funded by Medicare (Friel, 2010; Meadows *et al*., 2015).

It is not good enough to design service systems or provide interventions without due consideration to the context in which these services are to be delivered (Robert and Fulop, 2014). It is incumbent on service planners and providers to make the best use of local data to identify variation or disparity in service provision or treatment outcome attributable to contextual factors and build into these delivery systems enough safeguards to protect against such inequity. Healthcare could learn from the education sector which is moving, albeit fittingly, beyond the problem being the ‘poor’ student to understanding the student in context, establishing value from their perspective, and building the system accordingly (Dittmann and Stephens, 2017).

### Accountability and system co-design

Individuals have a valuable role to play in service planning and healthcare policy-making through meaningful and supported engagement and co-design in all levels and at all points of decision-making (Groves, 2010). Accountability frameworks should be designed accordingly, rather than being solely a transactional compact between funders and providers more honoured in the breech than the observance (Rosenberg and Salvador-Carulla, 2017). It is not realistic to expect meaningful shared-decision-making at the nano/micro-level of the clinical encounter when the overall context and system design at the meso- and macro-level is otherwise conditioned (Hoffmann *et al*., 2014). To do so transfers responsibility for making the system accountable from those who have the power and authority to those who, by design, are the least empowered within it, but paradoxically amongst the best placed to guide reforms (Berwick, 2002; Palmer *et al*., 2010). This creates not only a sense of individual frustration but from a planning perspective is a missed opportunity. Where co-design is systemically adopted, the benefits are cumulative and substantial (Iruin-Sanz *et al*., 2015; Palmer *et al*., 2019). Doing so should be the planning equivalent of an ‘always event’ (Lembitz and Clarke, 2009), and the same participatory and transparency principles should not be restricted to the governance of the day-to-day clinical workflow but built as a matter of course into all levels of the design, operation and adaption of healthcare systems (Holmes *et al*., 2016; Eyre *et al*., 2017).

This extends to the use of personal data. Measuring what is valued by patients and whether it is achieved, and reincorporating this into service development decisions is essential. Non-disclosive data on the planning and performance of health services funded through the public purse should be available for open scrutiny, reported at consistently meaningful aggregations – regional and sub-regional – and presented in a manner that is easily understood and relatable to the real-world, avoiding the use of obscurant metrics, or the bureaucratic tendency, well-meaning and benevolent as it may be, to confuse and conflate privacy and secrecy. As a result of this extractive rather than inclusive mentality to the use of personal data, most people have little if any idea whether their local health services are performing as they would wish or how they compare it to other localities in terms of value and cost (Chen, 2010). There are good examples that seek to change this bureaucratic myopia, the UK Spend Outcome Tools (SPOT) (Public Health England, 2019) and the European Public Health Observatories are two, there are just not enough of them and where they currently exist, they are often stand-alone offerings rather than substantively incorporated into key planning and decision processes. Such approaches do not generate improvements in the short term (Schang *et al*., 2014) but rather create the necessary conditions that enable open participatory engagement and collaboration to occur. Again, this is not a new idea and was raised in the IOM as one of the key enablers for overcoming the ‘quality chasm’; therefore, one must question who benefits most from the current state of secrecy.

## Conclusion

The relative impotence of planning when unsupported by clear change processes underpinned by implementation science means demand will continue to outpace supply as provision is constrained within an artificially constructed organic healthcare economy. This inhibits innovation under the guise of protection and governance and places a premium and priority on the providers return on investment and in doing so demotes value from the user's perspective to a secondary benefit. Unless altered, we will continue to invest in the wrong things, believing them to the right things, often plausible, but nearly always they create waste, not value. We apply the evidence-based dictum partially, choosing not to apply the same test to service planning and implementation that we do to treatments. Governance processes formalise the latter as a therapeutic good, and rightly so. Perhaps it is time to consider the application of a similarly constituted standard to the former that enables us to co-design, test and implement promising new models using local data, taking a person-centred and whole-of-community contextual approach, understanding healthcare is formally complex and ultimately recognise that it is the people who determine the value of services, not the funders. Without this, we will continue to build in demand failure and suffer the consequences.
